# Effect of laparoscopic sleeve gastrectomy vs laparoscopic Roux-en-Y gastric bypass on weight loss in Egyptian patients with morbid obesity

**DOI:** 10.1016/j.amsu.2021.103235

**Published:** 2022-01-04

**Authors:** Mohamad Baheeg, Saed A. Elgohary, Mohamed Tag-Eldin, Ahmed M.E. Hegab, Mahmoud S. Shehata, Esam M. Osman, Mohammed Eid, Yunus Abdurakhmanov, Mohamed Lamlom, Hazem A. Ali, Ahmed Elhawary, Momen Mahmoud, Mostafa Basiony, Yasien Mohammmed, Abdulkarim Hasan

**Affiliations:** a Department of Surgery, Faculty of Medicine, Al-Azhar University, Cairo, Egypt; b Department of Surgery, Faculty of Medicine, Al-Azhar University, Assuit, Egypt; c Department of Surgery, Faculty of Medicine, Al-Azhar University, Damietta, Egypt; d Department of Surgery, Faculty of Medicine, Peoples Russian Friendship University, Moscow, Russia; e Department of Psychiatry, Faculty of Medicine, Al-Azhar University, Cairo, Egypt; f Department of Anesthesia and Intensive Care, Faculty of Medicine, Al-Azhar University, Cairo, Egypt; g Primary Health Care Centers, Ministry of Health, Taba, Egypt; h Radiodiagnosis, Faculty of Medicine, Al-Azhar University, Cairo, Egypt; i Department of Pathology, Faculty of Medicine, Al-Azhar University, Cairo, Egypt

**Keywords:** Bariatric surgery, Laparoscopic surgery, Sleeve gastrectomy, Quality of life, Weight loss

## Abstract

**Background:**

Bariatric surgical operation is taken into consideration to be the handiest remedy for extreme obesity. Durability is the main requirement for the broad usage of bariatric surgery. According to several factors, the present work tries to match the SG and RYGB techniques.

**Methods:**

This is a retrospective work that studied 200 morbid obese patients randomized and categorized into two groups according to the treatment method: the laparoscopic sleeve gastrectomy (LSG) and LRYGB groups, within the period from 2014 to 2019 and matched weight dissipation, complications, quality of life, and adverse events.

**Results:**

BMI had a mean value of 39.66 ± 3.770 kg/m2 in the RYGB group versus 39.38 ± 3.648 kg/m2. No significant differences were found according to comorbidity, height, and weight. There was no significant difference between the study groups according to complications and morbidity—no recorded unexpected histopathology results in the excised LSG specimens.

**Conclusion:**

There was no significant change in weight dissipation, fluctuations in comorbidities, increase in Quality of Life (QoL), and complications for pathological obesity patients according to the treatment methods of laparoscopic SG (sleeve gastrectomy) and RYGB at 2-years postoperative follow-up.

## Highlights

• A study on 200 morbid obese patients of 2 groups; laparoscopic sleeve gastrectomy (LSG) and LRYGB.
• No significant difference between the study groups according to complications and morbidity.
• No significant difference in Quality of Life at 2-years postoperative follow-up.
• Histopathology results for the examined Sleeve gastrectomy specimen were unremarkable.
• Challenge of psychological or emotional changes should be considered.

## 1 Introduction

Obesity is the accumulation of overabundant fats [1] related to several chronic complications (e.g., T2DM (Type Two Diabetes Mellitus), hypertension, dyslipidemia, obstructive sleep apnea, osteoarthritis, and gastroesophageal reflux disease) [2]. Pathological obesity and its related complications are a big global challenge and an economic problem in many countries [3].

Although there are numerous treatment options for the morbidly obese by non-surgical weight dissipation methods based on the diet, exercise, drug administration and behavior, and some other therapeutic strategies, e.g., acupuncture [4], these methods cannot affect obesity-associated complications. In some patients, reverse reactions may be faced [5].

Bariatric surgical operation is very beneficial for morbid obesity therapy; this surgery was considered not only a metabolic but also a weight-dissipation operation [6]; it leads to an excellent durable steady weight-dissipation and decreases complications [7].

Bariatric surgical operation is advised for extreme obesity cases with a BMI (≥35 kg/m2) with at least one pathological situation (e.g., T2DM, hypertension, and obstructive sleep apnea) [8].

LRYGB (Laparoscopic Roux-en-Y gastric bypass) is the best and widely used for bariatric surgery due to its effectiveness and durability [9]. Still, this way needs a technicality and a long-term examination [10].

LRYGB has a higher loss in weight in comparison with some limited steps without clinically significant malabsorption [11]. It induces weight dissipation primarily by limiting food intake and dumping effect [12].

LSG (Laparoscopic Sleeve Gastrectomy) was considered to be the primary stage in a dual-step proceeding for high-obese patients’ treatment; now, it was considered as an independent way [13,14].

LSG is demonstrated by taking off about 80% of the side of the stomach in a perpendicular manner, leaving a residual tubular gastric pouch or sleeve [15]. The sleeve gastrectomy operation is technically handier to achieve and more rapid relative RYGB [16].

While one might assume that improved health, weight loss, and increased quality of life (QoL) would improve patients’ mood, a minority of patients may experience severe psychological complications, including depression, alcohol abuse, and suicidality, particularly after a period of 1–2 years post-surgery “honeymoon period.” [17].

In the present work, the prime goal was to match the SG and RYGB techniques according to weight dissipation, complications, life quality, and adverse events.

## 2 Material and methods

This is a retrospective work that studied 200 morbidly obese patients randomized and categorized into two groups according to the treatment method: the LSG and LRYGB groups.

The technique was illustrated with detail to all cases regarding probable complications and dietary plans after the operation. Informed written consent was obtained from each patient to be included in this work.

The study included cases age ranging from 18 to 65 with a BMI (Body Mass Index) more than 35 (BMI = weight (kg)/[height (m)]2), with at least one comorbidity (hypertension-dyslipidemia-obstructive sleep apnea-T2DM-arthritis) and previous failure of conservative treatment.

For Patients with BMI more than 60, we excluded psychiatric disorder, active gastric ulcer, active substance abuse, GERD (Severe Gastroesophageal Reflux Disease) with a large hiatal hernia, and previous bariatric surgery from the study.

Selected cases for histopathological examination.

The histopathological examination for the excised parts of LSG operations was performed for selected cases; the pathologists retrieved and studied the cases in this work.

The results of this study were reported in accordance with STROCCS reporting statements [18].

### 2.1 Interventions

**Fig. 1:**
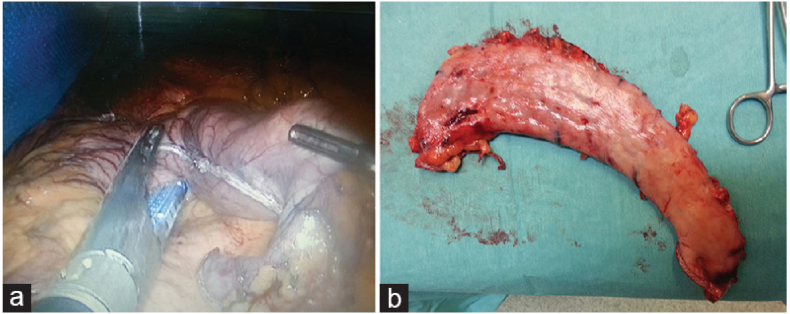
(a) Resection of the outer part of the stomach using *endo*-GI stapler during sleeve gastrectomy. (b) Excised part of the stomach after sleeve gastrectomy. GI, gastrointestinal.

LSG (Laparoscopic sleeve gastrectomy) technique:

0A 36 Fr bougie was applied over the lower curvature to adjust the gastric tube. Longitudinal amputation of the stomach was performed for about 4–6 cm per pecker of the pylorus to the corner of His. No supportive materials were used, and over-suturing of the basic line was performed only over the bleeding points (see Fig. 1 from a patient's medical file).

Specimens of LSG with any suspicious mucosal lesion were sent to histopathology laboratory for examination.

**Fig. 2:**
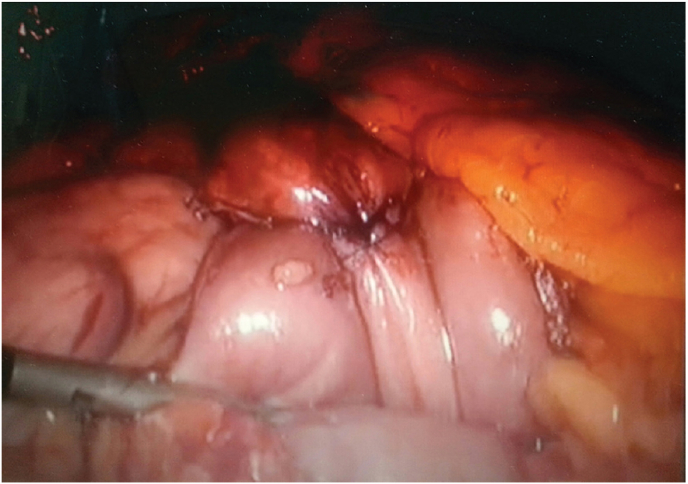
Gastrojejunostomy (pouch-jejunostomy) during RYGB.

RYGB (Laparoscopic Roux-en-Y gastric bypass) method:

An ante colic and antegastric RYGB become executed with a 150 cm alimentary limb with a linearly or circularly kink (25 mm) gastrojejunostomy in step with the desire of the surgeon. A 50-cm-lengthy biliopancreatic limb was elected (Fig. 2).

Methylene blue leak examination was routinely done during the operation, and a wide bore drain was applied near the staple line or anastomosis in both techniques.

After surgery, all the cases were periodically assayed on six weeks and after (3- 6-9-12-18-24) months, then annually.

All patients of the two groups underwent surgery under general anesthesia, premedicated with Metoclopramide 10 mg with Ranitidine 150 mg, pre -oxygenated with O2 100% for 5 min, Routinely ‘ramping’ in the 20° to 30° head-up position, then induction of anesthesia with propofol 1.5–2 mg/kg IBW(Ideal Body Weight), suxamethonium 1–1.5 mg/kg ABW(Actual Body Weight), Fentanyl 1 μg/kg ABW, lidocaine 1.5 mg/kg, intubated with rapid sequence induction using conventional laryngoscopy, McCoy laryngoscope (flexible tip blade) and bougie according to Mallampatti score (assessment of severity of difficult airway), maintenance of anesthesia with sevoflurane volatile agent, non-depolarizing muscle relaxant, fentanyl 50 mg/20 min, Ventilator settings were adjusted to maintain SPO2 between 94 and 100%and EtCO2 between 35 and 40 mmHg and. PEEP (Positive end expiratory pressure) of 5 cm H2O has been added to all patients, neuromuscular block has been reversed with Atropine 0.01 mg/kg and Neostigmine 0.04 mg/kg, after recovery pain controlled using multimodal analgesia.

### 2.2 Outcomes

The prime outcome of this work was weight dissipation that was recorded periodically.

The second outcomes included the change of obesity-related comorbidities as persisted, improved, or resolved and QoL based on GIQLI (Gastrointestinal Quality of Life Index) (consisting of 36 factors; ranging (0–144 points); each element scores from 4 to 0 points; the average record for normal persons is 125.8 points) [19] and the BAROS QoL (Bariatric Analysis and Reporting Outcome System QoL) score (consisting of 5 factors; record ranging (−3 to 3) points; for one factor the score range (1 to −1) and for the rest four factors ranging (0.5 to −0.5)) [20].

## 3 Results

A number of 200 cases were studied in this work during this period, according to the inclusion criteria. Of these 200 patients, 100 underwent RYGB and 100 with SG. Patients age with RYGB had an Average value of 41.30 ± 12.831 Vs. 42.60 ± 13.018 years.

Patients' sex shows that more than half in the RYGB groups were female 65 (65%) vs 52 (52%) in SG group. BMI had an average value of 39.66 ± 3.770 kg/m2 in RYGB group versus 39.38 ± 3.648 kg/m2. No significant differences were found according to comorbidity, height, and weight (see Table 1).

Fifty-one (51.0%) patients in the SG group and 49 (49.0%) in the RYGB group had HTN. At 24 months after postoperative, total recovery was recorded in 30 (58.8%) of 51 in the SG group relative to 34 (69.4%) of 49 in RYGB group, and there were no significant differences among the study groups (Table 2).

**Table 1:** Demographic data for the two studied groups.

	RYGB Group (n = 100)	SG Group (n = 100)	P Value
Age	41.30 ± 12.831	42.60 ± 13.018	0.945
Sex n(%)			
Male	35 (35.0%)	48 (48.0%)	0.085
Female	65 (65.0%)	52 (52.0%)	
Comorbidity			
HTN	49 (49.0%)	51 (51.0%)	0.888
Type 2 DM	54 (54.0%)	51 (51.0%)	0.777
Dyslipidemia	68 (68.0%)	65 (65.0%)	0.765
Obstructive Sleep Apnea	67 (67.0%)	68 (68.0%)	1.000
Arthritis	72 (72.0%)	67 (67.0%)	0.539
Height (cm)	173.91 ± 5.109	174.67 ± 5.461	0.290
Weight (kg)	119.69 ± 9.515	119.20 ± 9.700	0.879
BMI (kg/m^2^)	39.66 ± 3.770	39.38 ± 3.648	0.594

**Table 2:** Changes in Comorbidities at 2 yrs.

HTN	RYGB Group (n = 100)	SG Group (n = 100)	P Value
	49/100	51/100	
Remission	34 (69.4%)	30 (58.8%)	0.802
Improved	10 (20.4%)	12 (23.5%)	
Unchanged	3 (6.1%)	5 (9.8%)	
Worsened	2 (4.1%)	4 (7.8%)	
Type 2 DM	54/100	51/100	
Remission	44 (81.5%)	41 (80.4%)	0.903
Improved	3 (5.6%)	5 (9.8%)	
Unchanged	4 (7.4%)	3 (5.9%)	
Worsened	3 (5.6%)	2 (3.9%)	
Dyslipidemia	68/100	65/100	
Remission	38 (55.9%)	34 (52.3%)	0.258
Improved	24 (35.3%)	21 (32.3%)	
Unchanged	6 (8.8%)	10 (15.4%)	
Worsened	0 (0%)	0 (0%)	
Obstructive Sleep Apnea	67/100	68/100	
Remission	34 (50.7%)	36 (52.9%)	0.719
Improved	29 (43.3%)	30 (44.1%)	
Unchanged	2 (3.3%)	2 (2.9%)	
Worsened	2 (3.3%)	0 (0%)	
Arthritis	72/100	67/100	
Remission	36 (50.0%)	37 (55.2%)	0.312
Improved	24 (33.3%)	26 (38.8%)	
Unchanged	11 (15.3%)	4 (6.0%)	
Worsened	1 (1.4%)	0 (0%)	

At baseline, 51 (51.0%) of 100 in the SG group and 54 (54.0%) of 100 in the RYGB group had DM2. At 2 yrs. Postoperative, total recovery was recorded in 41 (80.4%) of 51 in SG group and 44 (81.5%) of 54 in RYGB group, with no statistical significance among the study groups regarding FBG (Fasting Blood Glucose) (Table 2).

Before surgery, regarding dyslipidemia, there was a 65 (65.0%) in the SG group and 68 (68%) in the RYGB group. Total recovery was recorded in 34 (52.3%) of 65 in the SG group versus 38 (55.9%) of 68 in the RYGB group 2 years postoperative (Table 2).

At baseline, 68 (68.0%) in SG group and 67 (67.0%) in RYGB group had Obstructive Sleep Apnea. At 24-month postoperative, A total recovery was reported in 36 (52.9%) of 68 in SG group vs 34 (50.7%) of 67in RYGB group with no statistical significance between the studied techniques (Table 2).

Arthritis was shown in 67 (67.0%) in SG group and 72 (72%) in RYGB group. Total recovery was reported value was 37 (55.2%) of 67 in the SG group in comparison with 36 (50.0%) of 72 in the RYGB group 24 postoperative with no statistical significance between the two studied techniques (Table 2).

Quality of Life (QoL) in the two studied groups within baseline and 24 months was significantly increased. There was no statistical significance between the two studies.

Groups on the GIQLI (SG, 118.15 points, vs RYGB, 120.35 points, and the BAROS QoL record (2.1 for SG vs. 1.96 points for RYGB) (Table 3).

**Table 3:** Quality-of-Life comparison for the study groups.

	Baseline		P Value	After 2 years		P Value
	RYGB Group (n = 100)	SG Group (n = 100)		RYGB Group (n = 100)	SG Group (n = 100)	
GIQLI	101.17 ± 8.788	99.37 ± 7.679	0.112	120.35 ± 8.999	118.15 ± 8.362	0.245
BAROS score	0.30 ± 0.359	0.30 ± 0.250	0.627	2.10 ± 0.220	1.96 ± 0.335	0.546

Table 4 show complications and Mortality rate in the two groups and there were no significant differences between them.

**Table 4:** Complications and death rate in the study groups.

Complication	RYGB Group (n = 100)	SG Group (n = 100)	*P* Value
Leak	2 (2.0%)	0 (0.0%)	0.497
Infection	5 (5.0%)	0 (0.0%)	0.059
Obstruction	1 (1.0%)	2 (2.0%)	1.000
Death	2 (2.0%)	0 (0.0%)	0.497
Morbidity			
Small bowel obstruction	3 (3.0%)	0 (0.0%)	0.246
Internal hernia	11 (11.0%)	0 (0.0%)	0.001*
Incisional hernia	3 (3.0%)	2 (2.0%)	1.000
Severe dumping	2 (2.0%)	0 (0.0%)	0.497
Insufficient weight loss	2 (2.0%)	6 (6.0%)	0.279

Histopathology examination was performed for the 8 cases of the LSG group; 3 cases showed normal (unremarkable) gastric features and 5 cases showed chronic gastritis. No malignancy was detected in all the examined specimens. All the received cases were shown clinical suspicion of abnormal mucosal lesions.

## 4 Discussion

Morbid obesity is a prime problem facing the globe which was considered the reason for a lot of comorbidities (e.g., cardiovascular disease, DM2, infertility, metabolic syndrome, and certain cancer types, giving rise to an increase in death rate) [21]. For severe obesity, bariatric surgical treatment is considered as the most successful management way, regarding the surgery type. Due to the variation in hormones and the patient's diet, this effect always takes place even before the beginning of weight dissipation [22].

Bariatric operation is the best way to manage morbid obesity patients. Till now, RYGB was considered the gold standard bariatric operation. However, SG was used recently with a growing rate regardless of the insufficiency of long-term efficacy. The LSG procedure is handier, faster, and might be more secure in comparison with RYGB. However, there are many more available references on the RYGB technique regarding long-time results of clinical and metabolic [23,24].

The auspicious short-time outcomes of LSG have somewhat changed from a two-step procedure to an independent bariatric method. LSG is considered to be less invasive, technically simpler, and operational handier compared with LRYGB. The long-time advantages of LSG include the absence of internal hernias, an intact gastrointestinal tract, and the shortage of malabsorption requiring lifetime observation of dietary status [25,26].

In the current study, the major aim was to compare SG and RYGB techniques regarding fat dissipation, decrease in comorbidities, increase in QoL, and negative events.

The patient's age in the group of RYGB in this study had a mean value of 41.30 ± 12.831 Vs 42.60 ± 13.018 years in LSG group. Patient's sex showed that more than half of patients in the RYGB groups were female 65 (65%) vs 52 (52%) in SG group. BMI had a mean value of 39.66 ± 3.770 kg/m2 in RYGB group versus 39.38 ± 3.648 kg/m2. No significant differences were found according to comorbidity, height, and weight.

In comparison with the study of Sherif [27], which was conducted on 434 patients, their BMI ranged (35 and 60 kg/m 2) with mean age of 42 ± 4.8 years, and 73% of them were women. Cases were assayed at (3–6 - 9 -12- 24) months. They were categorized into two random groups: the LSG group (214 cases) and the LRYGB group (220 patients), no statistical significance between the study groups was recorded regarding age, sex, BMI, and associated comorbidities.

However, another study by Yang et al. [28] reported that patients’ age in a group of RYGB had an average value of 40.4 ± 9.4 Vs. 41.4 ± 9.3 years in sleeve gastrectomy group, BMI had a mean value of 32.3 ± 2.4 kg/m2 in RYGB group versus 31.8 ± 3.0 kg/m2, the two groups had comparable anthropometric baseline, which includes age, sex, weight, BMI, waist perimeter, diabetes, and the use of medication.

The beneficial impacts of bariatric surgical operation on fat dissipation and comorbidities resulting from obesity are become trusted. In addition, these techniques can also be operated safely with a low death rate and morbidity risk [27]. There are few randomized controlled studies made a comparative study between two of the most usually used bariatric procedures - that is, LRYGB and LSG - with taking into account the weight dissipation and/or comorbidities resulting from obesity in the medium and long time [29].

At two years after surgery, the present study demonstrated that total recovery of HTN was seen in 30 (58.8%) of 51 in the SG group in comparison with 34 (69.4%) of 49 in the RYGB group, with no statistical significance between the studied groups. Total recovery of DM was seen in 41 (80.4%) of 51 in the SG group versus 44 (81.5%) of 54 in the RYGB group with no statistical significance in FBG (Fasting Blood Glucose) between the two groups. Preoperative, 65 (65.0%) in SG group and 68 (68%) in RYGB group had dyslipidemia, total recovery of this disease was recorded in 34 (52.3%) of 65 in the SG group vs 38 (55.9%) of 68 in RYGB group 24 months postoperative. At the same time, total recovery from sleep apnea was recorded in 36 (52.9%) of 68 in SG group in comparison with 34 (50.7%) of 67 in RYGB group with no significant differences. Total recovery of arthritis was seen in 37 (55.2%) of 67 in the SG group and 36 (50.0%) of 72 in the RYGB group 24-months postoperative, with no statistical significance between the studied groups.

According to the obesity-related comorbidities, Sherif [27] observed the recovery rate and development of hypertension, dyslipidemia, DM2, joint pain, obstructive sleep apnea syndrome, and GERD. There turned into a big development in comorbidities in the two groups 1 year postoperative. no statistical significance between the LSG group and LRYGB group regarding the remission of comorbidities or development rate except for the remission of GERD.

In agreement with our findings, similar results were reported by the study group of Maggard et al. [29] on the same subject, even with a lower BMI group, especially the rapid improvement in DM2 after the two operations.

Peterli et al. [23] reported in their study that at 5 yrs. Postoperative, total recovery of diabetes was reported in 16 (61.5%) of 26 in SG group and 19 (67.9%) of 28 in the RYGB group (absolute difference, −0.05%; 94% CI, −0.38%–0.27%; P > 0.98), total recovery of dyslipidemia was reported in 29 (42.6%) of 68 in SG group vs. 33 (62.3%) of 53 in the RYGB group 5 yrs. Postoperative.

Furthermore, in the present study, we found a significant increase in the QoL in the two groups between baseline and 2 years postoperative. Giving rise to the significant increase in GQOLI (SG: 118.15 points, & RYGB:120.35 points and the BAROS QoL score (SG: 2.1 vs RYGB:1.96 points).

In comparison with the study of Peterli et al. [23], in which QoL have a significant increase in the two groups among baseline and 5 years and for GQOLI (SG: 113.7 points, vs RYGB: 117.8 points; absolute difference, −4.34 points; 94.5% CI, −15.09 to 6.42 points; P = 0.41), there was no significance among the two studied groups.

Most importantly, as described by Zhang et al. [30], the trend of QoL appears parallel with %EWL, but the variations among both groups did not have a statistical significance at 5 years postoperative. The total score of the LSG and LRYGB groups are 1.32 ± 0.81 and 1.58 ± 0.72 (P = 0.18), respectively.

Finally, as regard complications and Mortality in the two groups, the current work concluded that there was no statistically significant differences among the studied groups according to complications and morbidity.

Consistent with what has been reported by Zhang et al. [29], the total complication rate was 15.62% (5/31) for LRYGB and 3.24% (1/31) for LSG (P > 0.05).

These results were in harmony with the study of Peterli et al. [23], wherein there has been no statistical significance in complications requiring surgical or endoscopic review within the first 5 yrs. Postoperative.

In regards to Salminen et al. [31], gastric bypass is accompanied by greater weight loss than sleeve gastrectomy over 5 year clinical trial. However, this weight loss wasn't statistically significant. In addition, Gastric bypass is associated with better control of hypertension than sleeve gastrectomy, but these results depended on the type of antihypertensive drug use.

In contrast with Salminen et al., laparoscopic sleeve gastrectomy was associated with more weight loss and better metabolic outcomes than laparoscopic Roux-en-Y gastric bypass over one-year intervals in the Asian population than in the Caucasian population according to Lakdawala et al. [32].

Patient may be referred for a radiological investigation to exclude complications in the early postoperative period or many months later. Radiologists are required to have an understanding of normal anatomy and the complications associated with evey kind of bariatric surgeries to interpret imaging studies correctly.

The Challenge of psychological or emotional changes should be taken into consideration for bariatric surgery. After the first few months of bariatric surgery, patients usually notice that they have settled into this “new normal,” but choosing a bariatric surgery is embarking on a lifelong process of learning about how to handle a new relationship with food and the body, with the need for multidisciplinary support [33].

Bariatric providers are encouraged to educate their patients about potential challenges before this surgery, inquire about the psychological complications afterward, validate the difficult experiences, and connect patients to required resources early in the adjustment period to prevent escalating problems.

The present study revealed only 8% of LSG operations were advised for histopathology examination according to clinician's suspicion of abnormal mucosa, as the followed policy in the hospital recommends selected histopathology examination rather than a routine examination of the SG specimens.

Routine histopathological study for gastrointestinal specimens, including LSG operations, occurs in many tertiary hospitals to confirm the samples and detect unexpected pathological abnormalities [24,34,35]. Histopathological data for SG specimens are insufficient to describe the common histopathological findings leading some authors to support the policy of routine histopathological examination of all SG specimens to detect any pathology that may have an impact on patient management [34,36]. Demirbas et al. [36] studied 253 patients who had undergone SG showing different pathologic findings; *H. pylori* positivity in 27% of patients, chronic active gastritis in 20.5%, chronic gastritis in 53.4%, and intestinal metaplasia in 2%, whereas unremarkable histopathologic findings were seen in 25.7% of patients.

In contract, this recommendation, Nowak et al. [37], and Al-Tokhy et al. [38] recommend that LSG specimens be subjects to gross pathologic (naked eye) examination in the vast majority of patients because most of the recorded histologic findings had no clinical impact on future treatment, with only a minority of samples being clinically significant.

It is difficult for us to put a recommendation for histopathology examination of LSG due to the minority of the examined cases.

## 5 Conclusion

Between patients with morbid obesity, there was no statistical significance in weight dissipation, comorbidities, increase quality of life, and negative events between LSG and LRYGB at 2 yrs of follow-up postoperative except for the occurrence of internal rupture on LRYGB group only. The Challenge of psychological or emotional changes (eg. feeling unexpected and isolating) should be considered.

## Ethical approval

Ethical approval was obtained from Al-Azhar University.

## Funding sources

This study did not receive any funding from public or private sectors.

## Author contribution

Study concept or design: MB, SAE, MT, ML, HAA, AE, MM, MBas, YM, AH. Data collection: MB, SAE, MT, AMEH, MSH, EMO, ME, YA, YM, AH. Data interpretation: MB, SAE, MT, YM, ML, HAA, MM, MBas, AH. Literature review: MB, MT, HAA, AE, ML, MBas, AH. Data analysis: MB, AE. Drafting of the paper: ALL. Editing of the paper: ALL. Manuscript revision: ALL.

## Registration of research studies

ClinicalTrials.gov number: NCT05145205.

## Guarantor

Dr. MB.

## Availability of data and materials

The data that support the findings of this study are available from the corresponding author upon reasonable request.

## Provenance and peer review

Not commissioned, externally peer-reviewed.

## Consent

NA.

## Declaration of competing interest

The authors declare no competing interests.
